# Estradiol-Mediated Axogenesis of Hypothalamic Neurons Requires ERK1/2 and Ryanodine Receptors-Dependent Intracellular Ca^2+^ Rise in Male Rats

**DOI:** 10.3389/fncel.2019.00122

**Published:** 2019-04-02

**Authors:** Lucas E. Cabrera Zapata, Mariana Bollo, María Julia Cambiasso

**Affiliations:** ^1^Instituto de Investigación Médica Mercedes y Martín Ferreyra, INIMEC-CONICET, Universidad Nacional de Córdoba, Córdoba, Argentina; ^2^Cátedra de Biología Celular, Facultad de Odontología, Universidad Nacional de Córdoba, Córdoba, Argentina

**Keywords:** hypothalamic neurons, axogenesis, estradiol, ERK1/2, Ca^2+^ signaling, ryanodine receptors

## Abstract

17β-estradiol (E2) induces axonal growth through extracellular signal-regulated kinase 1 and 2 (ERK1/2)-MAPK cascade in hypothalamic neurons of male rat embryos *in vitro*, but the mechanism that initiates these events is poorly understood. This study reports the intracellular Ca^2+^ increase that participates in the activation of ERK1/2 and axogenesis induced by E2. Hypothalamic neuron cultures were established from 16-day-old male rat embryos and fed with astroglia-conditioned media for 48 h. E2-induced ERK phosphorylation was completely abolished by a ryanodine receptor (RyR) inhibitor (ryanodine) and partially attenuated by an L-type voltage-gated Ca^2+^ channel (L-VGCC) blocker (nifedipine), an inositol-1,4,5-trisphosphate receptor (IP_3_R) inhibitor (2-APB), and a phospholipase C (PLC) inhibitor (U-73122). We also conducted Ca^2+^ imaging recording using primary cultured neurons. The results show that E2 rapidly induces an increase in cytosolic Ca^2+^, which often occurs in repetitive Ca^2+^ oscillations. This response was not observed in the absence of extracellular Ca^2+^ or with inhibitory ryanodine and was markedly reduced by nifedipine. E2-induced axonal growth was completely inhibited by ryanodine. In summary, the results suggest that Ca^2+^ mobilization from extracellular space as well as from the endoplasmic reticulum is necessary for E2-induced ERK1/2 activation and axogenesis. Understanding the mechanisms of brain estrogenic actions might contribute to develop novel estrogen-based therapies for neurodegenerative diseases.

## Introduction

For many years, estrogens have been recognized as one of the main orchestrators of the sexual differentiation of the brain, acting during critical periods of development to organize neural circuits in a way that determines the modulatory/activational effects of gonadal hormones in adulthood. Testosterone secreted by male rodent testes during development is aromatized in neurons to 17β-estradiol (E2), which displays multiple cellular processes that finally set the masculine phenotype (McCarthy, 2008; Wright et al., 2010). More recently, accumulating evidence indicates that E2 is not only a reproductive hormone but also a brain-derived neuroprotective factor, coordinating multiple signaling mechanisms that protect the brain from neurodegenerative diseases, affective disorders and cognitive decline (Arevalo et al., 2015). These beneficial actions in the brain have positioned estrogens as promising therapeutic compounds against different brain pathologies such as Parkinson and Alzheimer diseases, schizophrenia, multiple sclerosis, stroke, neuroinflammation, among others (Dye et al., 2012; Villa et al., 2016; McGregor et al., 2017; Giatti et al., 2018).

Classical estrogenic actions are mediated *via* intracellular estrogen receptors (ERs) that function as ligand-activated transcription factors to regulate the expression of estrogen-responsive genes. Additionally, estrogens generate a wide diversity of rapid “non-classical” effects, which occur in a range from some seconds to a few minutes *via* membrane-initiated mechanisms (Arevalo et al., 2012), including the triggering of Ca^2+^ signals (Beyer and Raab, 1998; Picotto et al., 1999; Wong et al., 2012), and the activation of several signaling pathways, such as phospholipase C (PLC)/inositol 1,4,5-trisphosphate (IP_3_) and diacylglycerol (Le Mellay et al., 1997; Chaban et al., 2004), nitric oxide synthase/nitric oxide (Kelly and Levin, 2001), adenylate cyclase/AMPc/protein kinase A (PKA; Beyer and Karolczak, 2000), phosphoinositide-3 kinase (PI3K; Garcia-Segura et al., 2010), PKC, and extracellular signal-regulated kinase 1 and 2 (ERK1/2) cascades (Wu et al., 2005).

Currently, it is known that E2 prevents cell death, promotes neuronal survival, and enhances neuritogenesis and synaptic plasticity in the brain (Carroll and Pike, 2008; Spence et al., 2013; Acaz-Fonseca et al., 2014; Khan et al., 2015; Lai et al., 2017; Céspedes Rubio et al., 2018). The induction of neurite outgrowth by E2 was first demonstrated by Toran-Allerand (1976, 1980) and Toran-Allerand et al. (1983) working with organotypic explant cultures of the preoptic area, hypothalamus, and cerebral cortex. This neuritogenic effect of the hormone was then observed in other brain regions, both directly related and unrelated with reproduction (Nishizuka and Arai, 1981; Reisert et al., 1987; Cambiasso et al., 1995; Murphy and Segal, 1996). Hypothalamic neurons *in vitro* undergo several intermediate stages of development from unpolarized to fully polarized cells (Díaz et al., 1992). Most of the neuritogenic effects of E2 were demonstrated in polarized neurons (stage III of development), which are characterized by the presence of axon (Díaz et al., 1992; Cambiasso et al., 1995). Previous studies from our laboratory have shown that E2 induces axonal growth through ERK1/2 activation in hypothalamic neurons of male embryos *in vitro*. Both axogenesis (Cambiasso and Carrer, 2001) and ERK1/2 activation (Gorosito and Cambiasso, 2008) mediated by the hormone are dependent on a membrane-initiated mechanism since E2-bovine serum albumin (BSA; a membrane-impermeable conjugate of E2) was as effective as free E2. Interestingly, decreasing intracellular Ca^2+^ by the Ca^2+^-chelator BAPTA-AM or blocking Ca^2+^-dependent PKC isoforms by Ro 32-0432 significantly decreased these E2 effects (Gorosito and Cambiasso, 2008). These findings strongly suggested an important role for Ca^2+^ in E2-induced ERK1/2 pathway activation and axonal growth; however, the results did not provide the mechanism of E2-induced Ca^2+^ signaling in hypothalamic neurons.

In this study, we found that E2 evoked activation of Ca^2+^ entry *via* L-type voltage-gated Ca^2+^ channels (L-VGCCs) and promoted Ca^2+^ release through ryanodine receptors (RyRs). This early Ca^2+^ response underlies E2-induced ERK1/2 activation and axogenesis in hypothalamic neurons. Altogether, these results bring new insights about the mechanism of brain estrogenic actions and might contribute to developing novel estrogen-based therapies for neurodegenerative diseases.

## Materials and Methods

### Animals and Cell Cultures

Embryos were obtained from pregnant Wistar rats at embryonic day 16 (E16). The day of vaginal plug was set as E0. Experimental procedures for handling and sacrificing animals were approved by the Animal Care and Use Committee at our institution (CICUAL-IMMF, INIMEC-CONICET-UNC; Córdoba, Argentina) and followed the NIH guidelines for care and use of laboratory animals. The minimum number of animals required was used for these experiments and suffering was minimized. Primary neuronal and astroglial cultures were prepared as previously described in Cambiasso et al. (2000). Pregnant rats were sacrificed by cervical dislocation under CO_2_ anesthesia, and the fetuses were dissected from the uterus. The male fetuses used for cultures were identified by visualization of the spermatic artery on the developing testes. Ventromedial hypothalamic and mesencephalic regions were dissected out and stripped off the meninges for primary neuronal and glial cultures, respectively. At E16, the axogenic effect of E2 is contingent on the presence of astroglia (Cambiasso et al., 1995) or astroglia-conditioned media from a target region (Cambiasso et al., 2000; Cambiasso and Carrer, 2001; Brito et al., 2004). The basal medium (BM) was (1:1) DMEM:Ham’s F12 Nutrient Mixture, supplemented with 0.043% l-alanyl-l-glutamine (GlutaMAX I), 0.15% glucose, 100 U/ml penicillin and 100 μg/ml streptomycin. All cultures were raised under phenol red-free conditions to avoid “estrogen-like effects” (Berthois et al., 1986). For neuronal cultures, the dissociated cell suspension was seeded on different supports pre-coated with 1 mg/ml poly-D-lysine depending on the experiment: 60 mm × 15 mm dishes (Corning Life Science, Tewksbury, MA, USA) for protein assays, 25 mm coverslips (Assistent, Germany) for Ca^2+^ imaging, and 12 mm coverslips (Assistent, Germany) for morphological studies.

### Western Blot

Hypothalamic neurons derived from male fetuses plated 1–2 h before were fed with astroglia-conditioned media for 2 days *in vitro* (DIV). After a 2 h washout period using BM, neuronal cultures were treated for 1 h with nifedipine (2 μM; Sigma-Aldrich, St. Louis, MO, USA), inhibitory ryanodine (50 μM; Santa Cruz Biotechnology, Santa Cruz, CA, USA), 2-APB (100 μM; Santa Cruz Biotechnology, Santa Cruz, CA, USA) or U-73122 (10 μM; Sigma-Aldrich, St. Louis, MO, USA), and then pulsed with 100 nM E2 (Sigma-Aldrich, St. Louis, MO, USA) for 15 min. Hormone concentration used was determined by dose dependence (1–100 nM) experiments previously performed by our group (Gorosito and Cambiasso, 2008; Gorosito et al., 2008). ERK phosphorylation was maximally increased after the application of 100 nM E2. This dose was then used for all further acute stimulation studies. We have used compounds at final concentrations that did not alter cell viability or morphology in control conditions.

After treatment, hypothalamic neurons were washed and harvested at 4°C in RIPA buffer [150 mM NaCl, 0.1% NP40, 0.5% sodium deoxycholate, 0.1% sodium dodecyl sulfate (SDS), 50 mM Tris, pH 7.5] with protease and phosphatase inhibitors (1 μg/ml aprotinin, 1 μg/ml leupeptin, 1 μg/ml pepstatin A, 5 μg/ml chymostatin, 5 μg/ml antipain, 100 μg/ml PMSF, 50 μM NaF, 10 μM Na_4_P_2_O_7_, and 1 mM NaVO_4_). Protein samples (20 μg/lane) were separated by 10% SDS-PAGE and transferred onto polyvinylidene fluoride membrane (Bio-Rad, Hercules, CA, USA). ERK1/2 phosphorylation was detected by using a rabbit monoclonal anti-phospho-p44/42 MAPK (Cell Signaling Technology, Danvers, MA, USA), which specifically detects both Thr202 and Tyr204 ERK phosphorylation forms of ERK [molecular masses (kDa) for ERK1 and ERK2 are 44 and 42, respectively]. Total ERK1/2 was detected by using a mouse anti-p44/42 MAPK (Cell Signaling Technology, Danvers, MA, USA). Secondary antibodies conjugated to horseradish peroxidase (Jackson, West Grove, PA, USA) were used for the detection by enhanced chemiluminescence on X-ray film. After incubation with the antibody against phospho-ERK1/2, blots were stripped and then re-probed with anti-total ERK1/2 to ensure equal protein loading. The resulting film samples were scanned and analyzed with an image analysis program (ImageJ; NIH, Bethesda, MD, USA). Data are presented as a ratio of phospho-ERK1/2/total-ERK1/2 of 3–4 different experiments (independent cultures) performed in duplicate.

### Ca^2+^ Imaging

After 2 DIV, hypothalamic neurons were treated (or not) with 50 μM ryanodine or 2 μM nifedipine for 1 h and incubated with 3 μM acetoxymethyl (AM) ester form of the organic Ca^2+^-dye Cal-520 (AAT Bioquest, Sunnyvale, CA, USA) for 30 min at 37°C in a Ca^2+^-containing HEPES buffered salt solution (Ca^2+^-HBSS) composed of (mM): 135 NaCl, 5.4 KCl, 2 CaCl_2_, 1 MgCl_2_, 10 HEPES, and 10 glucose; pH = 7.4 set with NaOH at RT. Following loading, neurons were washed twice with warm Ca^2+^-HBSS and were imaged in the same buffer. To analyze the participation of extracellular Ca^2+^, the cells were resuspended in an EGTA-containing buffer composed of (mM): 135 NaCl, 5 KCl, 0.5 CaCl_2_, 1.2 MgCl_2_, 5 HEPES, 14 NaHCO_3_, and 1 EGTA; pH = 7.4 set with NaOH at RT.

Imaging of cytosolic Ca^2+^ signals was performed using a 60× oil immersion objective of an Olympus IX81 inverted microscope [equipped with a Disk Spinning Unit (DSU), epifluorescence illumination (150 W Xenon Lamp), and a microprocessor], an ORCA AG (Hamamatsu) CCD camera and OSIS software. Frames were collected at a continuous rate of 2.5 per second during 5 min (790 frames). Cal-520 was excited at a wavelength of 492 nm, and emitted fluorescence was collected at 514 nm. E2 (100 nM) was added 30 s after starting the recording. 10 μM thapsigargin (tg) was added at 3 min of recording as a positive control of normal endoplasmic reticulum Ca^2+^ content. The fluorescence intensity of the Ca^2+^ indicator was analyzed using ImageJ (NIH, Bethesda, MD, USA) software and plotted as the change in fluorescence (ΔF) of 2 × 2 pixels divided by mean resting fluorescence [(Fo; ΔF/Fo)] over time. We measured both the peak fluorescence value and the integrated area under the ΔF/Fo curve with OriginPro 8 SR0 software (OriginLab Corporation, Northampton, MA, USA). The integrated area roughly corresponds to the total amount of Ca^2+^ released over the recording period.

### Immunocytochemical Staining

To analyze the effect of ryanodine in E2-stimulated axon growth without affecting the normal polarization of neurons, we performed the experiment after 1 DIV (stage III of development). After 2 h in absence of E2, the cultures were treated for 1 h with 50 μM ryanodine before the addition of 10 nM E2 for an additional 24 h. The hormone concentration used to study the neuritogenic effect of E2 was chosen based on previous studies of our laboratory (Gorosito et al., 2008; Scerbo et al., 2014).

After 2 DIV, neuronal cultures were fixed for 20 min with warm 4% paraformaldehyde in PBS containing 0.12 M sucrose and rinsed in PBS. Neurons were immunocytochemically stained with antibodies against β-tubulin class III (SDL.3D10). The details of the immunocytochemical procedure were as specified by Díaz et al. (1992). Briefly, the fixed cells were permeabilized in 0.2% Triton X-100 for 5 min at RT, preincubated with 5% BSA, incubated in mouse anti-β-tubulin class III (Sigma-Aldrich, St. Louis, MO, USA), rinsed in PBS, and finally incubated with appropriate biotinylated secondary antibody. Incubation with secondary antibody was followed by washing in PBS, incubation for 2 h in VECTASTAIN ABC immunoperoxidase reagent (Vector Laboratories, Burlingame, CA, USA), and a final reaction with 1.4 mM 3,3’-diaminobenzidine in phosphate buffer with H_2_O_2_. Coverslips were then dehydrated with ethanol, cleared with xylene, and mounted on glass slides for morphometric analysis. No immunostaining was detected when primary antibodies were replaced by 5% BSA.

### Morphometric Analysis

The morphometric analysis of stained neuronal cultures was performed on digitized video images using JAVA as an image processor (Jandel Inc., Richmond, CA, USA) controlled by a host computer. Images were acquired through an optic microscope (Carl Zeiss, Germany). Microscope slides were coded, and the person conducting the analysis was blind to the experimental group. All labeled cells that could be identified as one individual neuron were measured in random fields at 40× magnification. Neural processes were classified as minor processes or axons according to accepted morphological criteria (Dotti et al., 1988; Blanco et al., 1990; Díaz et al., 1992). Minor processes are two or three short neurites that emerge from the cell body; axons are much longer, unique, thin, and relatively uniform in diameter. Neurons were considered to have developed an axon if they showed one neurite three to five times longer than the rest (stage III of development). Soma area, length of minor processes, total axonal length, and the number of neurites per cell were recorded. At least 60 neurons were measured for every experimental condition in each culture; at least three separate cultures were made for every condition.

### Statistical Analysis

Data were statistically evaluated by one-way ANOVA, followed by Fisher’s Least Significant Difference (LSD) *post hoc* test (Statistica; StatSoft Inc., Tulsa, OK, USA) where *p* < 0.05 was considered statistically significant.

## Results

### E2-Induced ERK1/2 Activation Is Mainly Mediated by RyRs

**Figure 1:**
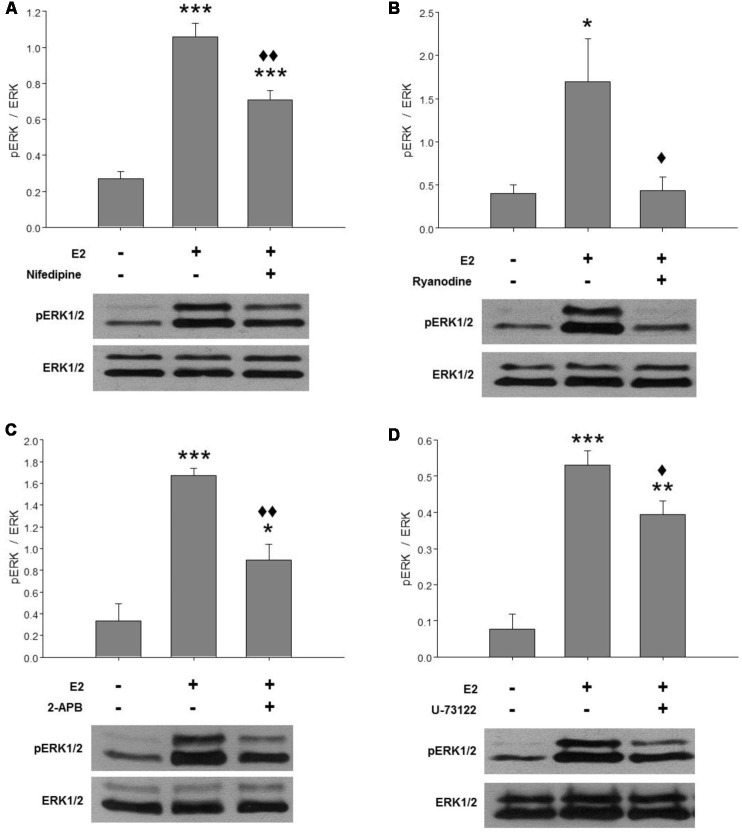
E2-induced extracellular signal-regulated kinase 1 and 2 (ERK1/2) activation depends on cytosolic Ca^2+^ increase mainly mediated by ryanodine receptors (RyRs). Effects of **(A)** 2 μM nifedipine, **(B)** 50 μM ryanodine, **(C)** 100 μM 2-APB or **(D)** 10 μM U-73122 on E2-induced ERK phosphorylation. After washing for 2 h, the cultures were treated with the inhibitors for 1 h and were then pulsed for 15 min with 17β-estradiol (E2) and harvested for Western blotting. Top: ratio of readings for pERK/ERK bands in arbitrary densitometric units. Bottom: examples of immunoblots showing a decrease of hormone-induced ERK phosphorylation in cultures pretreated with the inhibitors. Molecular masses (kDa) for ERK1 and ERK2 are 44 and 42, respectively. Blots shown are representative of the mean ± SEM of 3–4 different cultures. **(A)** Nifedipine: ANOVA *F*_(2,10)_ = 52.78; *p* ≤ 0.001. Least significant differences (LSDs) test indicated ***p < 0.001 vs. control and ^⧫⧫^*p* < 0.01 vs. E2. **(B)** Ryanodine: ANOVA *F*_(2,6)_ = 5.856; *p* = 0.04. LSDs test indicated *p = 0.05 vs. control and ^⧫^*p* = 0.05 vs. E2. **(C)** 2-APB: ANOVA *F*_(2,6)_ = 27.203; *p* ≤ 0.001. LSDs test indicated ***p < 0.001 and *p = 0.05 vs. control and ^⧫⧫^*p* = 0.01 vs. E2. **(D)** U-73122: ANOVA *F*_(2,6)_ = 34.891; *p* ≤ 0.001. LSDs test indicated ***p < 0.001 and **p = 0.01 vs. control and ^⧫^*p* ≤ 0.05 vs. E2.

Our previous results suggested that E2-induced ERK1/2 phosphorylation is Ca^2+^-dependent. Here, we further investigated the Ca^2+^ response involved in ERK activation mediated by E2. Hypothalamic cultures grown with E2 for 48 h were washed in BM for 2 h and pre-treated with specific compounds for 1 h before a pulse of E2 for 15 min. In agreement with previous reports, E2 induced a strong phosphorylation of ERK at 15 min (Figure 1). This effect was completely abolished by inhibitory ryanodine (Figure 1B) and partially attenuated by nifedipine (Figure 1A), IP_3_R inhibitor 2-APB (Figure 1C), and a PLC inhibitor U-73122 (Figure 1D).

### E2 Induces Rapid Ca^2+^ Increase Depending on Ca^2+^ Influx and RyRs

**Figure 2:**
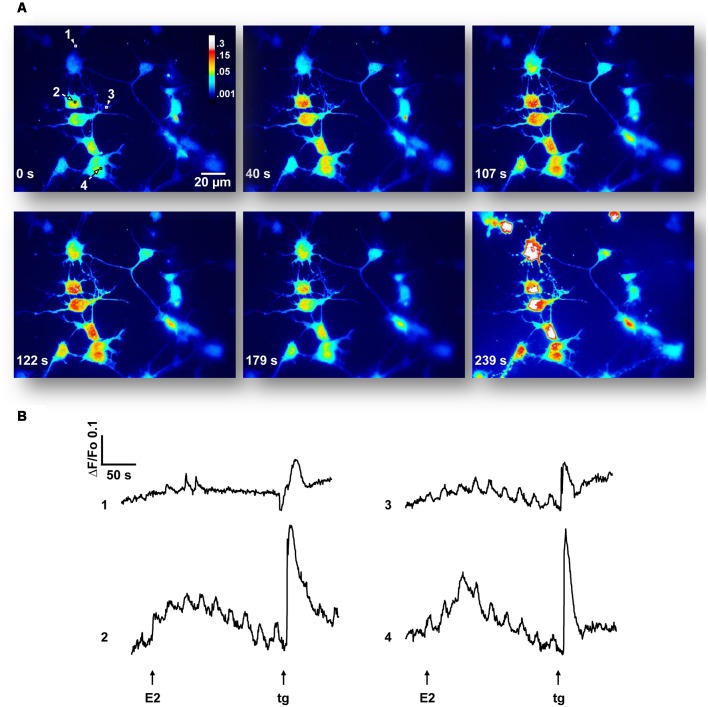
17β-estradiol (E2) induces Ca^2+^ oscillations. **(A)** Hypothalamic neurons were loaded with Cal-520 AM for 30 min at 37°C, maintained in a Ca^2+^-containing buffer (Ca^2+^-HBSS) and changes in cytosolic Ca^2+^ concentration were measured using confocal microscopy [Olympus IX81 inverted microscope equipped with a Disk Spinning Unit (DSU)]. Time series of Cal-520 pseudocolor images is shown, before and after the addition of E2 and thapsigargin (tg). Pseudocolor scale bar: 0.3–0.001 arbitrary units. Length scale bar: 20 μm. **(B)** Representative Ca^2+^ traces [regions of interest labeled 1–4 in **(A)**] plotted as changes over time in fluorescence intensity of the indicator (ΔF) respect to resting values (Fo). Arrows indicate the addition time of E2 (30 s) and tg (3 min). Data are from one representative experiment out of six independent experiments.

As E2-activation of the ERK1/2 signaling cascade depends on extracellular as well as intracellular Ca^2+^ stores, we decided to characterize the Ca^2+^ signal generated by the hormone. The addition of agonist to cell cultures, loaded with the indicator Cal-520 AM and imaged in a Ca^2+^-containing buffer, induced fluorescence changes that were observed in neuronal soma as well as in minor processes (Figure 2). Seventeen out of 76 neurons (22.4%) from six independent experiments imaged in Ca^2+^-HBSS responded to E2 pulses within 15–100 s of treatment (average = 46.8 ± 6.60 s). These Ca^2+^ events often occur in repetitive oscillations, which display interspike intervals of 21.58 ± 8.83 s (9 out of 17 neurons). The amplitude in terms of ΔF/Fo was 0.154 ± 0.01. The total amount of Ca^2+^ mobilized, measured as the integrated area under the curve (AUC) for all E2-generated peaks, was 4.69 ± 1.03, which was 90.6% with respect to the control [remained endoplasmic reticulum Ca^2+^ content released by tg, a sarco/endoplasmic reticulum Ca^2+^-ATPase (SERCA) inhibitor].

**Figure 3:**
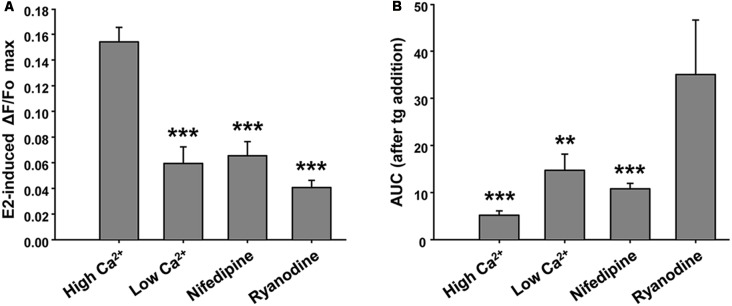
Ryanodine, external Ca^2+^ and nifedipine modulate E2-induced Ca^2+^ increase. **(A)** Mean values of maximal ΔF/Fo for Ca^2+^ mobilized by 17β-estradiol (E2) in hypothalamic neurons recorded either in the presence or absence of extracellular Ca^2+^ (Ca^2+^-HBSS/EGTA-containing buffer, named High Ca^2+^/Low Ca^2+^), or after a pre-incubation period (1 h) with ryanodine or nifedipine. ANOVA *F*_(3,41)_ = 22.94; *p* ≤ 0.001. LSDs test indicated ***p < 0.001 vs. Ca^2+^-HBSS. **(B)** Mean values of integrated area under ΔF/Fo curve (AUC) after thapsigargin (tg) addition during Ca^2+^ imaging [same conditions as **(A)**]. ANOVA *F*_(3,26)_ = 9.18; *p* ≤ 0.001. LSDs test indicated ***p < 0.001 and **p = 0.01 vs. ryanodine. Bars represent mean ± SEM; *n* = 4–6 different cultures.

This E2-induced Ca^2+^ signal was not observed in the absence of extracellular Ca^2+^. Moreover, we found that pre-incubation of neuronal cultures with inhibitory ryanodine suppressed E2-evoked Ca^2+^ release (Figure 3A). Importantly, under these conditions, the tg was able to mobilize amounts of Ca^2+^, measured as AUC, of 14.69 ± 3.45 (EGTA-containing buffer) and 35.05 ± 11.54 (ryanodine pre-incubation), five and nine times greater, respectively, than the amounts of Ca^2+^ mobilized by tg in E2-induced control neurons (Figure 3B). Moreover, nifedipine reduced the E2-induced Ca^2+^ increase more than 50% (ΔF/Fo = 0.065 ± 0.011; and AUC = 10.779 ± 1.159, *n* = 4), which strongly suggests the participation of L-VGCCs in this signal (Figures 3A,B). Representative Ca^2+^ traces plotted as ΔF/Fo vs. time for EGTA, ryanodine, and nifedipine conditions are provided in Supplementary Figure S1.

Taken together, these results indicate that both Ca^2+^ influx and mobilization from intracellular stores contribute to the response.

### E2-Induced Axonal Growth Depends on Ca^2+^ Signal Generated by RyRs

**Figure 4:**
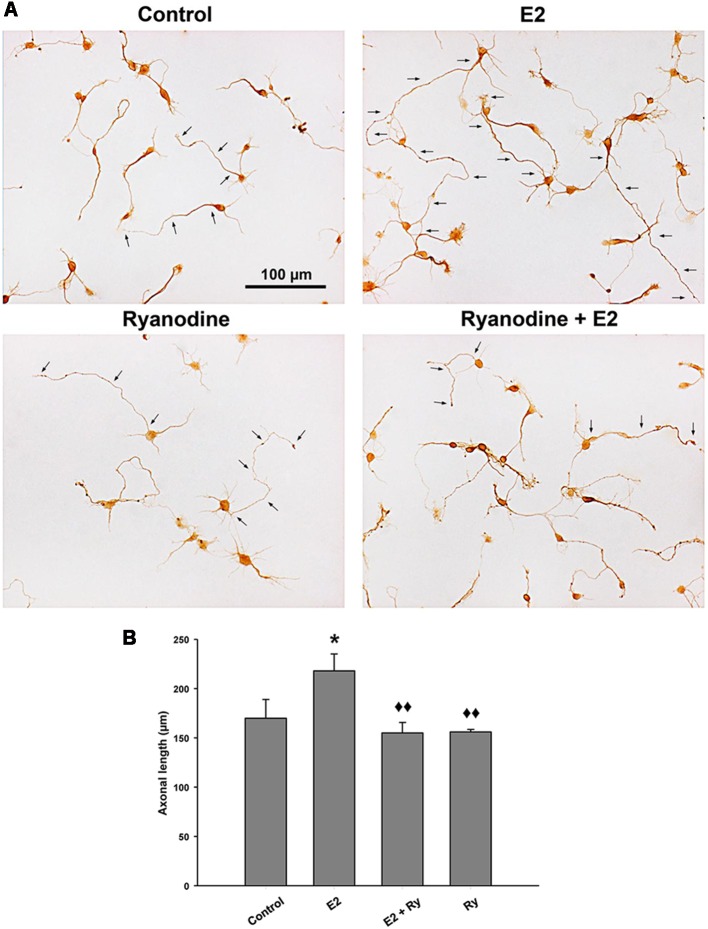
RyR activity is required to the E2-induced axogenesis. **(A)** Representative images of male hypothalamic neurons cultured for 48 h with (E2) or without (control) 10 nM 17β-estradiol in combination or not with 50 μM ryanodine (Ry) for the last 24 h of incubation (arrows indicate the axons of some neurons). **(B)** Mean of axonal length for each condition in **(A)**. ANOVA *F*_(3,12)_ = 4.51; *p* = 0.02. LSDs test indicated *p = 0.05 vs. control and ^⧫⧫^*p* = 0.01 vs. E2. Data represent the mean ± SEM; *n* = 4 independent cultures. Scale bar: 100 μm.

Finally, we tested whether the RyR-induced Ca^2+^ response is part of the signaling cascade that mediates the axogenic effect of E2. Neurons grew under the following conditions: in the presence and absence of E2 and pretreated with inhibitory ryanodine. After these treatments, the cells were grown for an additional 24 h period with (E2) or without E2 (control). In agreement with previous results, the morphometric analysis indicated that neurons grown under hormonal treatment show longer axons than neurons in control conditions without E2 (Figure 4A). Remarkably, blocking RyRs with ryanodine completely inhibited the E2-induced axogenesis (Figure 4B). Moreover, no significant differences were observed in the number of primary neurites, length of minor processes, or soma area per neuron resulting from E2 or ryanodine treatment (Table 1), confirming that the hormonal effect is restricted to axonal growth (Díaz et al., 1992; Cambiasso et al., 1995, 2000; Brito et al., 2004).

**Table 1:** Number of primary neurites, soma area, and length of minor processes of male hypothalamic neurons grown with or without 17β-estradiol (E2) in 3 combination or not with ryanodine.

Variable	Treatment			
	Control	E2	E2 + Ryanodine	Ryanodine
N° of neurites	4.2 ± 0.2	4.4 ± 0.2	4.3 ± 0.4	4.2 ± 0.2
Soma area (μm^2^)	118.9 ± 13.2	116.6 ± 4.5	119.5 ± 10.0	126.2 ± 9.0
Minor processes length (μm)	107.2 ± 7.7	115.6 ± 8.9	94.0 ± 11.5	92.6 ± 5.8

*Data represent the mean ± SEM; n = 4 independent cultures*.

## Discussion

In the present study, results from ERK1/2 phosphorylation, Ca^2+^ imaging and neuronal growth consistently pointed to RyRs as the Ca^2+^ channels necessary to mediate activation of the MAPK/ERK pathway and the final axogenic effect induced by E2. Inhibitory ryanodine completely blocked E2-mediated Ca^2+^ transients, ERK1/2 phosphorylation and axonal outgrowth, which provides valuable evidence to propose that E2 mobilizes endoplasmic reticulum stores of Ca^2+^ through RyRs to activate the signaling cascades that finally affect the axonal elongation of hypothalamic neurons.

In regard to the impact of functional RyRs on cellular response, Dr Hidalgo’s group showed that BDNF-induced neural plasticity requires functional RyRs activated by the Ca^2+^-induced Ca^2+^ release (CICR) mechanism to evoke the larger Ca^2+^ signaling needed to maintain changes during long-term memory storage (Adasme et al., 2011). CICR is a positive feedback mechanism by which cytoplasmic Ca^2+^ stimulates Ca^2+^ release from the endoplasmic reticulum through RyRs or IP_3_Rs (Bezprozvanny et al., 1991; Berridge et al., 2003; Seo et al., 2015). Moreover, several studies have indicated a regulatory role for estrogens on RyRs activity, for instance, in the human eccrine sweat gland cell line NCL-SG3 (Muchekehu and Harvey, 2008), ventricular myocytes (Yan et al., 2011), detrusor smooth muscle cells (Hristov et al., 2017), and dorsal root ganglion neurons (Ferrari et al., 2016; Khomula et al., 2017). Interestingly, Zhao X. et al. (2005) proposed, in a neuroblastoma cell line, an E2-mediated mechanism starting at the plasma membrane, by which rapid Ca^2+^ signaling potentiates the transcription of genes normally regulated by estrogens; RyRs, IP_3_Rs, and N-VGCCs, but not L-VGCCs, were involved in the process. Our results clearly show that RyRs are indispensable Ca^2+^ channels involved in the non-classical signaling events produced by E2 to generate axonal growth in hypothalamic neurons. However, RyRs also require a previous small increase in cytosolic Ca^2+^ from resting levels to activate and release Ca^2+^ by CICR (Hidalgo et al., 2005; Lanner et al., 2010). Since we found that removing extracellular Ca^2+^ or blocking the membrane channels L-VGCCs prevented the Ca^2+^ signaling induced by E2 and that inhibiting L-VGCCs also reduced ERK1/2 phosphorylation modulated by the steroid, we postulate that E2 initially induces a Ca^2+^ influx in hypothalamic neurons *via* L-VGCCs that then enables RyRs opening to generate the final and complete Ca^2+^ signaling event (Hidalgo, 2005; Calin-Jageman and Lee, 2008). Besides L-VGCCs, our results show that the PLC/IP_3_Rs system is involved in E2-induced ERK1/2 activation, since both 2-APB and U-73122 used as blockers of IP_3_Rs and PLC, respectively, produced a significant reduction in phosphorylation levels of the kinases in the presence of the hormone. The activation of PLC and Ca^2+^ release *via* IP_3_Rs induced by estrogens has been previously reported in different cellular systems (Chaban et al., 2004; Fricke et al., 2007).

L-VGCCs are the major route of Ca^2+^ entry into neurons and the most profusely studied and best characterized VGCC type by far, as they play a predominant role in the brain (Striessnig et al., 2014; Vega-Vela et al., 2017). Several studies report that E2 is able to modulate L-VGCCs activity (Bulayeva et al., 2005; Sarkar et al., 2008; Farkas et al., 2012; Feng et al., 2013). Wu et al. (2005) and Zhao L. et al. (2005) indicated that E2 induced rapid Ca^2+^ influx through L-VGCCs, which was required to activate the Src/ERK/CREB/Bcl-2 signaling pathway and finally mediated neuroprotective and neurotrophic responses in rat hippocampal and cortical neurons. The generation of this intracellular Ca^2+^ increase and the downstream activation of ERK depend on the presence of ERs in the membrane of rat hippocampal neurons (Wu et al., 2011). These membrane ER-expressing neurons represented 29% of the cultured cells and all of them co-expressed L-VGCCs. Consistently, our data from Ca^2+^ imaging experiments indicated that approximately 22% of hypothalamic neurons responded to E2.

Although previously it has been reported that E2 induces L-VGCCs-mediated Ca^2+^ influx, the question about how the hormone activates L-VGCCs remains open. Sarkar et al. (2008) reported that E2 potentiated the activity of L-VGCCs by directly binding to specific sites in the channel, independently of ERs. On the other hand, PI3K signaling cascade has been proposed as a candidate to link membrane ER activation with L-VGCCs aperture (Simoncini et al., 2000; Quignard et al., 2001; Wu et al., 2005), although our preceding work blocking PI3K with LY-294,002 did not prevent the axogenic effect of E2 in hypothalamic neurons (Gorosito and Cambiasso, 2008). It is important to note that we have previously reported the expression of ERα on the cell-surface of embryonic hypothalamic neurons (Gorosito et al., 2008), and that the membrane-impermeable E2-albumin construct (E2-BSA) was as effective as free E2 to generate ERK1/2 phosphorylation (Gorosito and Cambiasso, 2008) and axonal elongation (Cambiasso and Carrer, 2001), evidence that altogether indicate these processes respond to a membrane-initiated ERα-mediated mechanism.

Another question that arises is what other elements lead from the RyRs-mediated Ca^2+^ release to ERK1/2 activation. In Gorosito and Cambiasso (2008) we reported that PKC but not PKA nor CaMKII is required in the E2-induced MAPK-ERK pathway activation, since an inhibitor with specificity for the PKC Ca^2+^-dependent α and βI isoforms, Ro 32-0432, attenuated E2-modulated ERK1/2 phosphorylation and prevented the axogenic effect of the hormone. PKC activation by E2 has been found in different cell types, including breast cancer cells, hepatocytes, and cortical and hypothalamic neurons (Marino et al., 1998; Boyan et al., 2003; Cordey et al., 2003; Qiu et al., 2003). Ca^2+^-dependent PKC activation can then induce ERK1/2 phosphorylation *via* Src/Ras signaling (Cullen and Lockyer, 2002; Brandt et al., 2003; Roskoski, 2005).

Our results show that E2-induced cytosolic Ca^2+^ often increases as repetitive oscillations. This is in agreement with previous work demonstrating E2-induced intracellular Ca^2+^ oscillations that involved internal stores and PKA and PLC activity in neurons of the arcuate nucleus (Fricke et al., 2007). It is well known that the frequency of Ca^2+^ oscillation may depend on Ca^2+^ influx into the cell (Sneyd et al., 2004), SERCA activity (Falcke et al., 2003) and oscillating cytoplasmic IP_3_ concentration (Sneyd et al., 2006). Our data suggest that the frequency of Ca^2+^ oscillations in hypothalamic neurons stimulated by E2 mainly depends on Ca^2+^ entry. Leaving aside its modulation, our results allow us to speculate that the characteristic frequency encodes information to regulate the cellular response (axonal growth) mediated by the hormone (Dolmetsch et al., 1998).

In conclusion, we have provided new insights into the non-classical mechanisms triggered by estrogens and its axogenic effect in male rat hypothalamic neurons. The hormone induces ERK1/2 activation in a Ca^2+^-dependent manner. RyRs inhibition abolished this activation as well as axonal growth. The oscillatory Ca^2+^ signal generated by E2 required functional RyRs and L-VGCCs. This early Ca^2+^ response that underlies E2-induced RyRs and MAPK/ERK activation may transmit a finely tuned message into a neuronal development program, reflecting the need for tight control of a critical event during sexual differentiation of the male brain. The conjunction of XY genotype with adequate estrogen exposure levels at the time of hypothalamic neuronal differentiation may induce the growth of axons towards their appropriate targets. A complete and detailed understanding of the intracellular signaling mechanisms and neuronal processes mediated by estrogens will allow to improve current estrogen-based therapies, such as hormone replacement therapy in postmenopausal women, as well as to develop novel treatments to prevent and/or alleviate neurological pathologies based on its widely proven neuritogenic and neuroprotective effects.

## Author Contributions

LCZ, MB and MC made the conception and design of research, interpreted results of experiments, wrote the manuscript. LCZ performed all experiments and analyzed the data. LCZ and MB prepared the figures. MB and MC edited and revised the manuscript. MC drafted the manuscript.

## Conflict of Interest Statement

The authors declare that the research was conducted in the absence of any commercial or financial relationships that could be construed as a potential conflict of interest.
