# Supplementary figures and images for: Estradiol-Mediated Axogenesis of Hypothalamic Neurons Requires ERK1/2 and Ryanodine Receptors-Dependent Intracellular Ca2+ Rise in Male Rats
Source: Front Cell Neurosci. 2019 Apr 2;13:122. doi: 10.3389/fncel.2019.00122 (PMC6454002; doi:10.3389/fncel.2019.00122)

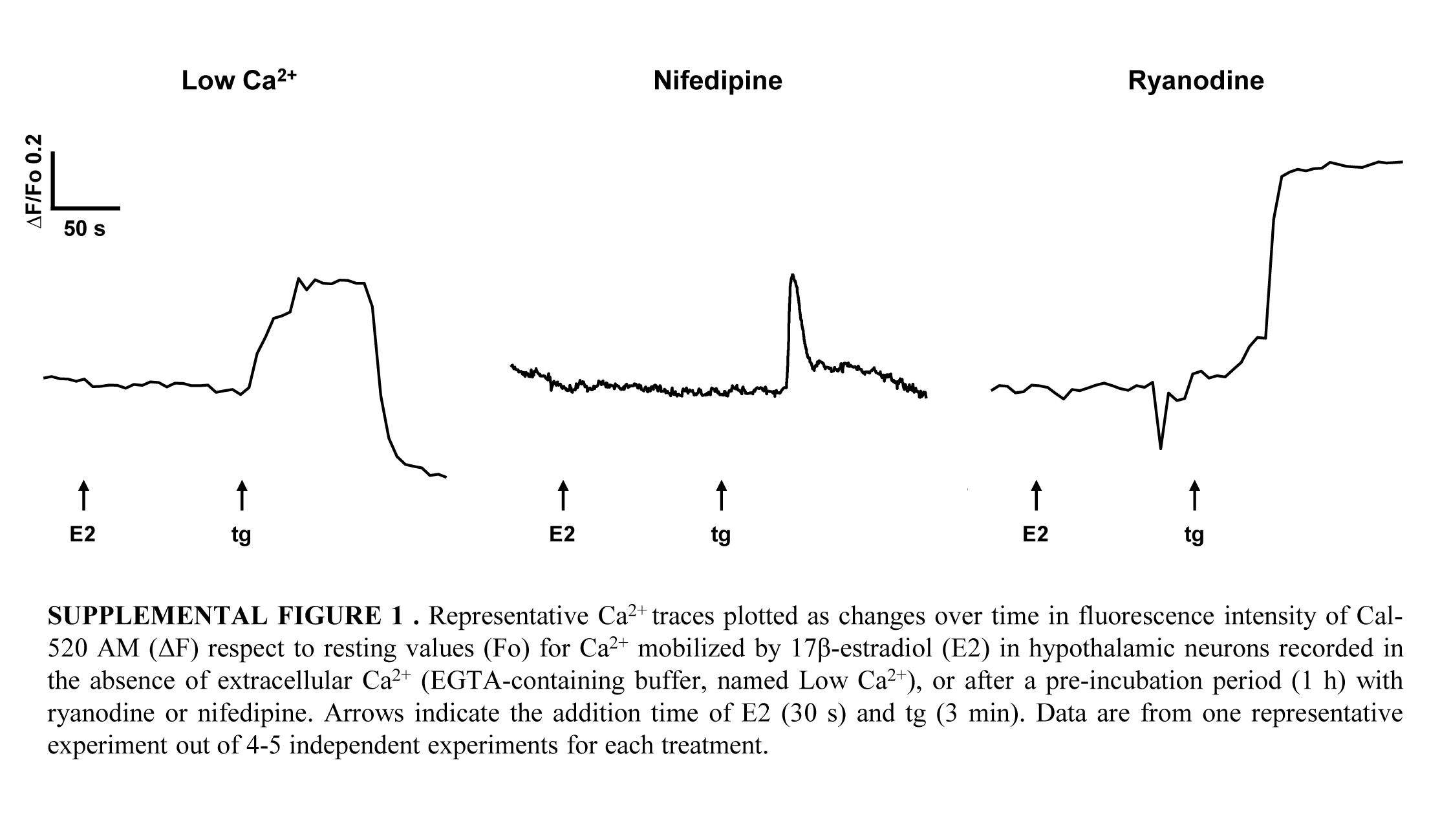

Supplement: Supplementary file 1 [file Image_1.TIF]
